# The Role of ClpV in the Physiology and Pathogenicity of *Xanthomonas citri* subsp. *citri* Strain zlm1908

**DOI:** 10.3390/microorganisms12122536

**Published:** 2024-12-09

**Authors:** Ya Li, Zilin Wu, Dengyan Liu, Kexin Cong, Jiajun Dai, Wenjie Xu, Yingtong Ke, Xinyi He

**Affiliations:** College of Coastal Agricultural Science, Guangdong Ocean University, Zhanjiang 524088, China; 2112204011@stu.gdou.edu.cn (Z.W.); 296723380@stu.gdou.edu.cn (D.L.); 2112304076@stu.gdou.edu.cn (K.C.); daiddd@stu.gdou.edu.cn (J.D.); xuwenjie@stu.gdou.edu.cn (W.X.); 1148933603@stu.gdou.edu.cn (Y.K.); 2941159730@stu.gdou.edu.cn (X.H.)

**Keywords:** *Xanthomonas citri* subsp. *citri*, T6SS, *clpV*, physiologic function, pathogenicity

## Abstract

*Xanthomonas citri* subsp. *citri* (*Xcc*) is a Gram-negative bacterium responsible for citrus canker, a significant threat to citrus crops. ClpV is a critical protein in the type VI secretion system (T6SS) as an ATPase involved in bacterial motility, adhesion, and pathogenesis to the host for some pathogenic bacteria. In order to investigate the function of *clpV* gene in *Xcc*, the *clpV*-deletion strain Δ*clpV* was constructed, its biological properties were evaluated, and the differences in gene expression levels between the wild-type strain and Δ*clpV* were analyzed by transcriptomics. The results exhibited significantly reduced biofilm formation, extracellular polysaccharide synthesis, and swarming motility in Δ*clpV* compared to the wild-type strain. Although the *clpV*-deletion did not significantly affect bacterial growth or pathogenicity in terms of disease symptoms on citrus leaves, the mutant showed increased sensitivity to environmental stresses (NaCl, SDS, and H_2_O_2_) and antibiotics (β-lactams and aminoglycosides). Transcriptome analysis revealed that *clpV*-deletion altered the expression of motility-related genes and the efflux pump gene *mexH*. Our findings underscore the importance of ClpV in maintaining biofilm integrity and suggest a multifaceted role in adaptive strategies of *Xcc*, positioning ClpV as a potential target for mitigating citrus canker disease.

## 1. Introduction

*Xanthomonas citri* subsp. *citri* (*Xcc*) is a Gram-negative bacterial pathogen that causes citrus canker disease, one of the most serious diseases affecting citrus crops, resulting in significant economic losses for the citrus industry [1]. This pathogen infects host plants through stomata and wounds, invading the intercellular spaces in the apoplast [2]. It produces erumpent, corky necrotic lesions often surrounded by a chlorotic halo on leaves, young stems, and fruits. As the lesions develop, they lead to defoliation, rupture of the leaf epidermis, and premature fruit drop in citrus plants [3].

Currently, *Xcc* has been extensively studied to elucidate the molecular mechanisms underlying its pathogenesis and interactions with host plants. The type VI secretion system (T6SS) is recognized as a contact-dependent secretion machinery capable of directly injecting toxins into both other bacteria and eukaryotic cells. Several studies have indicated that T6SS serves as a virulence factor in many plant-pathogenic bacteria, including *Burkholderia glumae*, *Xanthomonas oryzae* pv. *oryzae*, and *Ralstonia solanacearum* [4,5,6]. T6SS delivers antimicrobial or cytotoxic effector proteins to recipient cells through direct contact, exerting toxic effects and serving as an important weapon for most Gram-negative pathogenic bacteria in both environmental competition and host infection [7]. Despite its significance, information about the functions of T6SS in plant pathogens remains limited. T6SS consists of a trans-envelope complex that docks a contractile tail composed of an inner tube made of stacked Hcp (hemolysin co-regulated protein) hexamers, topped by the VgrG (valine-glycine repeat protein G) syringe, and surrounded by a sheath formed from polymerized TssB and TssC subunits, along with Hcp, IcmF (intracellular multiplication protein), VgrG, and ClpV. ClpV is a critical protein in this machinery as an ATPase that forms a hexameric complex, providing the energy required for the secretion of T6SS substrates [8]. Li et al. [9] demonstrated that the release of Hcp1 depends on ATP hydrolysis facilitated by the ClpV domain in *Pseudomonas aeruginosa*, indicating that the role of ClpV in Hcp1 secretion is energy-dependent.

The *clpV* (also named *tssH*) deletion mutation in *Xanthomonas phaseoli* pv. *manihotis* has confirmed that the disruption of *clpV* reduces the resistance to H_2_O_2_-induced oxidative stress [10]. In *Agrobacterium tumefaciens*, *clpV* deletion resulted in the inability to secrete Hcp into the supernatant, although Hcp secretion was partially restored in the complementary strain [11]. ClpV has been identified as a virulence factor in *Pseudomonas plecoglossicida* [12,13]. ClpV3 of the H3-type T6SS (H3-T6SS) has been recognized as a multiple virulence factor in *P. aeruginosa*, where the intracellular concentration of the secondary messenger cAMP was reduced in the PA01 (Δ*clpV3*) strain [9].

As the core components and effector functions of T6SS, the function of ClpV has still been unknown in *Xcc*. In this study, we constructed a *clpV*-deletion strain and studied its biological characteristics, then analyzed the differences in gene expression levels between the wild-type and *clpV*-deletion strain by transcriptomics.

## 2. Materials and Methods

### 2.1. Bacterial Strains, Plasmids, and Experimental Plants

The bacterial strains and plasmids utilized in this study are detailed in Table 1. *Xcc* zlm1908 was isolated in August 2019 from the leaf of *Citrus sinensis* ‘hongjiang’ suffering from citrus canker and stored in our laboratory. The strain was cultured at 30 °C in Luria–Bertani (LB) medium. *Escherichia coli* DH5α and β2163 strains were cultured at 37 °C in LB medium. Healthy 3-year-old citrus trees (*C. sinensis* ‘hongjiang’) were obtained from Zhanjiang Huacheng Agriculture Development Company (Chikan district, Zhanjiang City, China) and cultivated in 20 cm × 30 cm pots in a greenhouse. The leaves of *C. sinensis* ‘hongjiang’, known to be susceptible to citrus canker, were used for *Xcc* infection experiments.

### 2.2. Cloning and Sequencing of clpV Gene from Xcc zlm1908

Genomic DNA from *Xcc* zlm1908 (WT) was extracted using the Steadypure Universal Genomic DNA Extraction Kit (AG Accurate Biotechnology, Changsha, China). The primers *clpV*-CF and *clpV*-CR (Table 2) were employed to amplify the *clpV* gene. PCR was conducted under the following conditions: an initial denaturation at 96 °C for 4 min, followed by 30 cycles of 95 °C for 60 s, 55 °C for 45 s, and 72 °C for 45 s, with a final extension at 72 °C for 7 min. The PCR products were purified using the Takara MiniBEST DNA Fragment Purification Kit Ver. 4.0 (Takara, Dalian, China). The purified PCR product was then ligated into the pMD18-T plasmid and sequenced by Sangon Biological Engineering Technology & Services Co., Ltd. (Sangon, Guangzhou, China).

### 2.3. Construction of the clpV Deletion Mutant

For the construction of Δ*clpV*, upstream fragments A and downstream fragments B of the *clpV* gene were amplified using primers *clpV*-UF/*clpV*-UR and *clpV*-DF/*clpV*-DR, respectively. The fragments containing the upstream region and the downstream region of *clpV* were obtained, respectively. An overlap sequence of 15 bp allowed the amplification of a fragment containing a deletion from nucleotides 51 to 1432 of the clpV ORF through a second PCR with the primer pairs *clpV*-UF/*clpV*-DR. The two fragments with overlapping sequences were then used as templates for subsequent PCR amplification.

The PCR product was ligated into the suicide vector pLP12, and the recombinant vector pLP12-clpV was extracted and transformed into *Escherichia coli* β2163. Single crossover mutant strains were generated by conjugal transfer of the recombinant vector into the WT genome. Deletion mutant strains were screened on 0.3% D-glucose LB plates. Primer pair *clpV*-UF/*clpV*-DR was employed for PCR identification to confirm the successful construction of Δ*clpV*. For the construction of the complementing plasmid for the *clpV*-deletion, the *clpV* fragment was amplified using specific primers and ligated into the pBAD33CM-rp4 vector. The recombinant vector *clpV*-pBAD33CM-rp4 was transformed into *E. coli* DH5α. The plasmid DNA was isolated and transformed into Δ*clpV* to produce the complementary strain. Positive clones were selected and sequenced to confirm the successful construction of C-*clpV*.

### 2.4. Characteristic Analysis of ΔclpV

#### 2.4.1. Genetic Stability Analysis of Δ*clpV*

The Δ*clpV* and C-*clpV* were continuously cultured for 50 generations in LB plates at 30 °C for 12 h. PCR identification using primers *clpV*-UF/*clpV*-DR was performed to confirm the stability of each generation of Δ*clpV* and C-*clpV*.

#### 2.4.2. Observation of Morphological Characteristics

Strains WT, Δ*clpV*, and C-*clpV* were cultured in LB plates at 30 °C for 12 h. The colonies were suspended in sterile water to prepare bacterial suspension for scanning electron microscope. The samples were viewed and photographed using a Hitachi XA-650 scanning electron microscope (SEM, Tokyo, Japan).

#### 2.4.3. Growth Curve in Medium and on Leaves

Strains WT, Δ*clpV*, and C-*clpV* were cultured in LB media. Bacterial solutions (OD_600_ = 0.2) were inoculated into 20 mL LB media at a 1:100 ratio and incubated with shaking at 180 rpm and 30 °C for 60 h. The OD_600_ value was measured every 4 h to generate the growth curve. For the growth curve on leaves, 40 μL of each bacterial solution (OD_600_ = 0.2) was infiltrated into *C. sinensis* ‘hongjiang’ leaves using a needleless syringe [14]. Leaf samples were collected at different time points (0–6 days post inoculation). Leaf disks (1 cm^2^ in diameter) near the infection site were excised with a cork borer and rinsed with 75% alcohol and then with ddH_2_O three times. The disks were ground in 1 mL ddH_2_O using a sterilized mortar. A 100 μL aliquot of the sample homogenate was serially diluted and cultured on LB plates at 30 °C for 72 h. The bacterial colonies were counted to evaluate the growth of each strain. The experiments were repeated independently three times.

#### 2.4.4. Biofilm Formation and Adhesion Assay

Biofilm formation was assessed in glass tubes using the method of Yan and Wang [15]. A 5 mL adjusted bacterial solution (OD_600_ = 0.2) of WT, Δ*clpV*, and C-*clpV* was, respectively, transferred to glass tubes and incubated statically at 30 °C for 24, 48, and 72 h. After incubation, the culture medium was removed, and the tubes were washed three times with ddH_2_O and air-dried. Then, 5 mL of 0.1% crystal violet was added to each tube for 15 min. The tubes were rinsed with ddH_2_O and dried, and biofilm formation was visualized by the crystal violet stain. The dye was dissolved in 95% alcohol, and absorbance was measured at 570 nm for quantification.

On the leaf surface, 20 μL of each bacterial suspension (OD_600_ = 0.2) was incubated on *C. sinensis* ‘hongjiang’ leaves at 30 °C for 24 h in a moist environment. Bacterial adhesion to the leaves was measured using the same crystal violet method. Each assay was performed independently three times with nine inoculation sites for each strain.

#### 2.4.5. Swarming Motility Analysis

For swarming motility analysis, 2 μL of bacterial suspension (OD_600_ = 0.2) was added to LB media supplemented with 0.3% agar in Petri dishes, following the method of Gottig et al. [16]. The inoculated plates were incubated at 30 °C for 72 h. After incubation, the motility zone was measured by calculating the diameter of the swarming area. Three spots were tested for each strain in every experiment, and the experiments were independently repeated three times.

#### 2.4.6. Sensitivity Analysis to Stress and Antibiotics

The stress factors used included 0.15 M NaCl, 0.01% sodium dextran sulfate (SDS), and 0.01 mM H_2_O_2_. Antibiotics β-lactams (Cefotaxime and Ceftriaxone), aminoglycosides (kanamycin and neomycin sulfate), and sulfonamides (Trimethoprim and sulfafurazole) were prepared at 10 mg/mL, filtered (0.22 μm), and stored in 4 °C. Serial 2-fold dilutions were made to concentrations of 1024 to 0.25 μg/mL. WT, Δ*clpV*, and C-*clpV* strains were cultured in LB media at 30 °C for 12 h, adjusting OD_600_ to 0.2. The bacterial solutions were inoculated into the stress factors and diluted antibiotics solutions (1:1 ratio) and cultured at 180 rpm and 30 °C for 24 h. The OD_600_ values were measured using a spectrophotometer, with LB media serving as a control.

#### 2.4.7. Extracellular Polysaccharide (EPS) Formation Ability

According to the method of Montenegro et al. [10], 2 mL of bacterial suspension (OD_600_ = 0.2) from WT, Δ*clpV*, and C-*clpV* strains was incubated in a 50 mL centrifuge tube at 30 °C for 48 h. After incubation, three volumes of anhydrous ethanol were added, and the mixture was centrifuged at −4 °C for 10 min. The supernatant was discarded, and the precipitate was washed with 70% anhydrous ethanol to extract soluble polysaccharides. The soluble polysaccharide was then dissolved in 1 mL of 5 M NaOH. Following this, 1 mL of 5% phenol and 2 mL of concentrated sulfuric acid were sequentially added. The samples were treated in darkness for 10 min, vortexed for 30 s, and then bathed in water at 25 °C for 20 min. Absorbance was measured at 490 nm to determine the content of soluble exopolysaccharides. Each experiment was repeated three times to ensure reproducibility.

#### 2.4.8. Extracellular Enzyme Activity

In this experiment, 2 μL of bacterial suspension (OD_600_ = 0.2) from WT, Δ*clpV*, and C-*clpV* strains were inoculated onto LB plates supplemented with 1% soluble starch, 2% β-mannose, and 1% carboxymethyl cellulose, respectively. After culturing at 30 °C for 72 h, extracellular enzyme activities, including amylase, β-mannanase, and cellulase, were assessed. For amylase activity, 15 mL of Lugol’s iodine solution was added to the plates and stained for 15 min. The diameter of the transparent hydrolysis zone (D) and the colony diameter (d) were measured, and the ratio D/d was calculated. To evaluate β-mannanase activity, 20 mL of 0.1% Congo red staining solution was added, followed by rinsing with 1 M NaCl twice for 10 min each. The diameter of the yellow hydrolysis zone was recorded. The cellulase activity was assessed using the same method as for β-mannanase. Three colonies were used for each stain in every experiment, with each experiment repeated three times for accuracy.

#### 2.4.9. Pathogenicity Assay

Pathogenicity assays were conducted according to the method described by Guo et al. [17]. The 3-year-old *C. sinensis* ‘hongjiang’ trees were grown in a quarantine greenhouse facility at Guangdong Ocean University. Fully expanded leaves were rinsed sequentially with 75% alcohol and distilled water (ddH_2_O) and then air-dried in preparation for the pathogenicity assay. The strains WT, Δ*clpV*, and C-*clpV* were incubated in a shaking incubator at 180 rpm and 30 °C. Samples were collected at 24 h, and the different bacterial concentrations (OD_600_ = 0.1, 0.2, 0.5, 0.7, and 0.9) were adjusted.

For the pathogenicity assay assessing different bacterial concentrations, a 40 μL aliquot of bacterial suspension was inoculated onto leaf surfaces by either puncturing the leaves along both sides of the main vein with sterilized insect needles (pinprick) or by infiltrating the suspension directly with a sterile syringe (infiltration). All inoculated leaves were maintained in a moist environment using wet, degreased cotton for 3 d. Symptoms and disease progression were monitored and recorded phenotypically when a pinhole-sized lesion appeared. Each strain was inoculated on at least five leaves per experiment, and the experiments were repeated three times.

### 2.5. Transcriptome Sequencing

#### 2.5.1. Transcriptome

WT and Δ*clpV* strains were cultured in LB liquid medium at 30 °C for 12 h. Samples were concentrated by centrifugation at 4 °C, then immediately frozen in liquid nitrogen and stored at −80 °C. Total RNA was extracted from the frozen samples using the RNA Prep Pure Cell/Bacteria Kit (Tiangen Biotech, Beijing, China) for transcriptome sequencing. Strand-specific RNA-seq libraries were prepared using the NEBNext^®^ Ultra™ Directional RNA Library Prep Kit for Illumina^®^ (NEB, Ipswich, MA, USA). Following cluster generation, the library preparations were sequenced on an Illumina HiSeq platform (Illumina, San Diego, CA, USA) at Beijing Novogene Bioinformatics Technology Co., Ltd. (Novogene, Beijing, China).

#### 2.5.2. qRT-PCR Validation

The cDNA was synthesized using the EasyScript One-Step gDNA Removal and cDNA Synthesis SuperMix (Takara) for reverse transcription of total RNA. Quantitative real-time PCR (qRT-PCR) was conducted with SYBR Green Pro Taq HS Premix II (Takara) on the CFX Connect™ Optics Module Real-Time System (Bio-Rad, Hercules, CA, USA). All data were analyzed using 16S rDNA as an internal reference for qRT-PCR, and relative expression levels were calculated as fold change values using the 2^−ΔΔCt^ method [18]. A total of four genes identified from the RNA-seq data were assessed by qRT-PCR. Specific primer pairs for each gene were designed and are listed in Table 2. Experiments were performed in triplicates. The Pearson correlation coefficient between the qRT-PCR and RNA-seq results was calculated using IBM SPSS 22.0 software (SPSS, New York, NY, USA).

### 2.6. Statistical Analysis

All data are expressed as the mean ± SEM. Statistical analysis was performed using Student’s *t*-test or one-way analysis of variance (ANOVA), followed by the post hoc test (least significant difference test and Duncan’s multiple range test). Differences were considered to be significant if *p* < 0.05.

## 3. Results

### 3.1. Cloning of clpV Gene and Construction of ΔclpV

**Figure 1:**
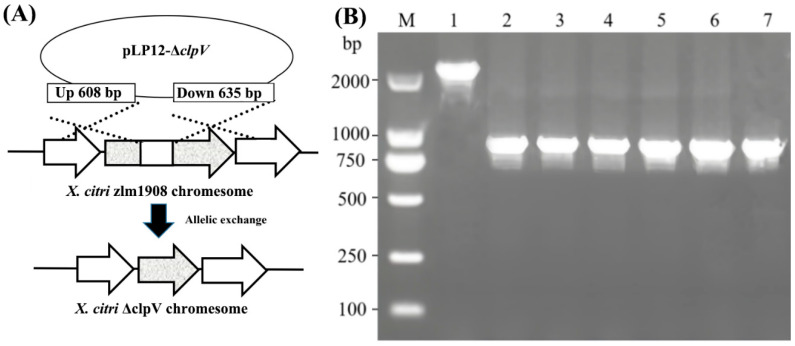
Construction of Δ*clpV* mutant strain of *X. citri*. subsp. *citri* strain zlm1908. (**A**) The strategy of *clpV* gene was knocked out by allele exchange in *Xcc*. (**B**) The construction of the deletion strain was confirmed by PCR amplification. M: DL2000 marker; lane 1: *Xcc* wild type; lane 2: Δ*clpV* mutant strain; lane 3: 10 generations of Δ*clpV*; lane 4: 20 generations of Δ*clpV*; lane 5: 30 generations of Δ*clpV*; lane 6: 40 generations of Δ*clpV*; lane 7: 50 generations of Δ*clpV*.

A 1569 bp fragment was obtained by PCR using the primer pair *clpV*-CF/CR. Sequence analysis in GenBank confirmed that *clpV* was cloned correctly. The upstream fragment A and downstream fragment B of *clpV* gene, measuring 408 bp and 435 bp, respectively, were obtained. The AB fragment, which is 828 bp in length, was amplified through overlapping PCR using fragments A and B as templates. The deletion mutant strain Δ*clpV* was constructed by overlapping amplification, removing base pairs 51 to 1432 of the *clpV* gene using forward and reverse screening methods. The deletion mutant Δ*clpV* was confirmed by PCR amplification, yielding the expected band size (Figure 1A). The complementation of Δ*clpV* was achieved using forward screening methods, resulting in the successful construction of C-*clpV*, as confirmed by the screening of positive clones and PCR (Figure 1B).

### 3.2. Characterization of ΔclpV

#### 3.2.1. Genetic Stability of Δ*clpV*

The stable inheritance of WT, Δ*clpV*, and C-*clpV* were confirmed by continuous 50 generations. The results show that 2209 bp and 828 bp fragments were, respectively, obtained by PCR for WT and Δ*clpV* for all of colonies of 1 to 50 generations, indicating all the tested strains exhibited stable inheritance (Figure 1B).

#### 3.2.2. Morphological Characteristics

**Figure 2:**
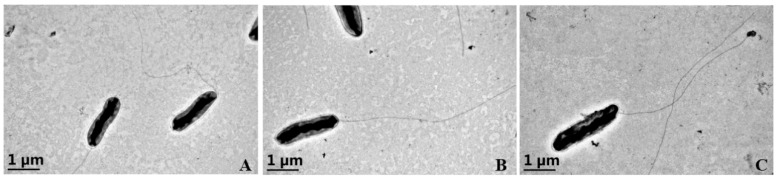
Morphological characteristics of *X. citri* subsp. *citri* strain zlm1908 (**A**), Δ*clpV* (**B**), and C-*clpV* (**C**) (SEM, 25,000× magnification).

The morphology of WT, Δ*clpV*, and C-*clpV* was observed using scanning electron microscopy. All strains displayed a short rod shape, rounded at both ends, with a single polar flagellum and encapsulation. These observations indicate that there are no significant morphological differences among the three strains (Figure 2).

#### 3.2.3. Growth Curve

**Figure 3:**
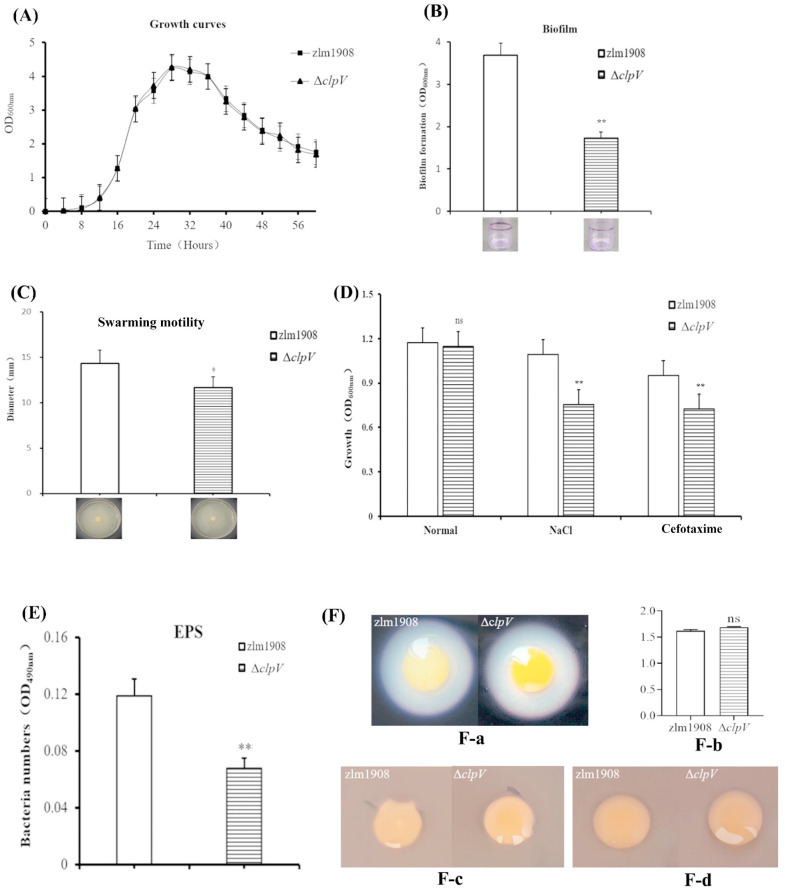
The biological characteristics of Δ*clpV* mutant compared with *Xcc* wild type. (**A**) The growth curve of *Xcc* zlm1908 and Δ*clpV* in LB culture medium for 60 h; (**B**) biofilm formation; (**C**) swarming motilities; (**D**) susceptibility to various stress conditions; (**E**) the production of EPS; (**F**) extracellular enzyme activity: (**F-a**) extracellular amylase, (**F-b**) extracellular amylase hydrolysis circle diameter/colony diameter, (**F-c**) β-mannanase, and (**F-d**) cellulase. ns: no significant difference (*p* > 0.05) The experimental results were averaged over the three replicates, and the error bars represent standard deviations (SD). * *p* < 0.05; ** *p* < 0.001; ns, not significant (Student’s *t*-test) (n = 3 independent experiments).

Strains WT, Δ*clpV*, and C-*clpV* exhibited similar growth curves in LB media and on leaves (Figure 3A). In LB media, the exponential growth phase ranged from 8 to 30 h, with the stationary phase beginning at 32 h for all strains. The in-frame deletion of *clpV* did not affect the growth of *Xcc* in the medium. Regarding the growth curves on leaves, changes of the *Xcc* population near the inoculated dot were monitored from 0 to 6 d post inoculation (dpi) for WT, Δ*clpV*, and C-*clpV*. The results indicate that all strains had colonized the leaves. From 2 dpi to 4 dpi, the population of Δ*clpV* was maximal, having significant difference compared to those of WT and C-*clpV*. These results indicate that the deletion of the *clpV* gene had reduced colonization on the surface of *C. sinensis* ‘hongjiang’ leaves.

#### 3.2.4. Biofilm Formation and Adhesion Ability

Biofilm formation was measured at OD_570_ in glass tubes and on leaf surfaces using the crystal violet ammonium oxalate assay. In the glass tubes, the results indicate that the OD_570_ values for WT, C-*clpV*, and Δ*clpV* gradually increased with the incubation times. The Δ*clpV* strain exhibited a significantly reduced biofilm formation at 24, 48, and 72 h (Figure 3B). On the leaf surface, the adherence ability of Δ*clpV* significantly decreased compared to both WT and C-*clpV* (*p* < 0.05).

#### 3.2.5. Swarming Motility

The cell motility of WT, Δ*clpV*, and C-*clpV* was evaluated on 0.3% agar plates at 30 °C for 72 h. The results show that the swarming circle diameters were 37.5 ± 1.5 mm for WT, 15.4 ± 0.75 mm for Δ*clpV*, and 35.8 ± 0.9 mm for C-*clpV* (Figure 3C). A significant difference in swarming diameter was observed between Δ*clpV* versus both WT and C-*clpV* (*p* < 0.01).

#### 3.2.6. Sensitivity to Stress and Antibiotics

The sensitivity of WT, Δ*clpV*, and C-*clpV* was tested under stress conditions involving 0.3 M NaCl, 0.01% SDS, 0.01 mM H_2_O_2_, and diluted antibiotics. The OD_600_ value for Δ*clpV* was the lowest among the three strains when subjected to 0.3 M NaCl, 0.01% SDS, and 0.01 mM H_2_O_2_ stress, with significant differences observed between Δ*clpV* versus both WT and C-*clpV* (*p* < 0.05). The deletion of *clpV* exhibited lower resistance to these stresses. The minimal inhibitory concentration (MIC) of Δ*clpV* for β-lactam antibiotics (cefotaxime and ceftriaxone) and aminoglycosides (kanamycin and neomycin sulfate) was two times higher than that of WT and C-*clpV*. However, the MIC of Δ*clpV* for sulfonamides (trimethoprim and sulfafurazole) was similar to that of WT and C-*clpV* (Figure 3D). These results indicate that the deletion of *clpV* leads to a reduction in sensitivity to some antibiotics.

#### 3.2.7. Extracellular Polysaccharides Formation

Extracellular polysaccharides (EPS), recognized as a key virulence factor, are closely associated with the infection and pathogenicity of bacteria. In this study, the EPS values were measured as 0.068 ± 0.02 for Δ*clpV*, 0.119 ± 0.01 for WT, and 0.124 ± 0.05 for C-*clpV* (Figure 3E). A significant difference was observed between Δ*clpV* versus both WT and C-*clpV* (*p* < 0.01). These results indicate that the deletion of *clpV* leads to a decrease in EPS synthesis.

#### 3.2.8. Extracellular Enzyme

The enzyme activities of extracellular amylase, β-mannanase, and cellulase in Δ*clpV*, WT, and C-*clpV* were evaluated by measuring the diameter of the hydrolysis circle. The results indicate that the ability to hydrolyze β-mannan and cellulose was absent in all three strains (Figure 3F). No significant differences in extracellular amylase activity were found between Δ*clpV* versus both WT and C-*clpV*.

#### 3.2.9. Pathogenicity

**Figure 4:**
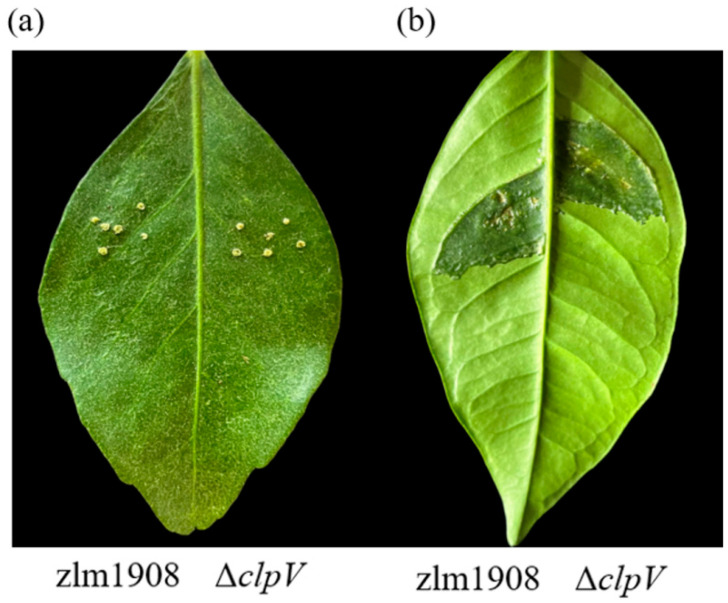
Disease symptoms of the leaves at 15 days after inoculated with an OD_600_ of 0.2 in *C. sinensis* ‘hongjiang’. (**a**) pinprick inoculation (**b**) infiltration inoculation.

The pathogenicity of Δ*clpV*, WT, and C-*clpV* was assessed on 3-year-old *C. sinensis* ‘hongjiang’. The leaves were inoculated by the pinprick or infiltration using different bacterial concentrations, and the most severe disease symptoms were observed at 15 dpi. When leaves were inoculated with the different concentrations of WT, Δ*clpV*, and C-*clpV* (OD_600_ = 0.1, 0.2, 0.5, 0.7, and 0.9) after 24 h of incubation, there were no significant differences observed in the disease symptoms on leaves inoculated with the same method and concentration among the Δ*clpV*, WT, and C-*clpV* strains (Figure 4 and Table 3).

### 3.3. Transcriptome Analysis

#### 3.3.1. Identification and Analysis of Differentially Expresses Genes (DEGS)

The constructed library was sequenced using the Illumina platform, yielding 7,596,458 uniquely mapped reads for WT and 7,433,469 for Δ*clpV*. A significance threshold of *p* ≤ 0.05 and |log_2_(Fold change)| ≥ 1.0 were applied to identify differentially expressed genes (DEGs) between WT and Δ*clpV*. The analysis revealed a total of 76 unigenes, comprising 34 upregulated and 42 downregulated genes. The DEGs were primarily associated with metabolism, iron receptor protein transport, bacterial motility, and stress resistance (Supplementary Table S1).

#### 3.3.2. Gene Ontology Functional Classification of DEGS

**Figure 5:**
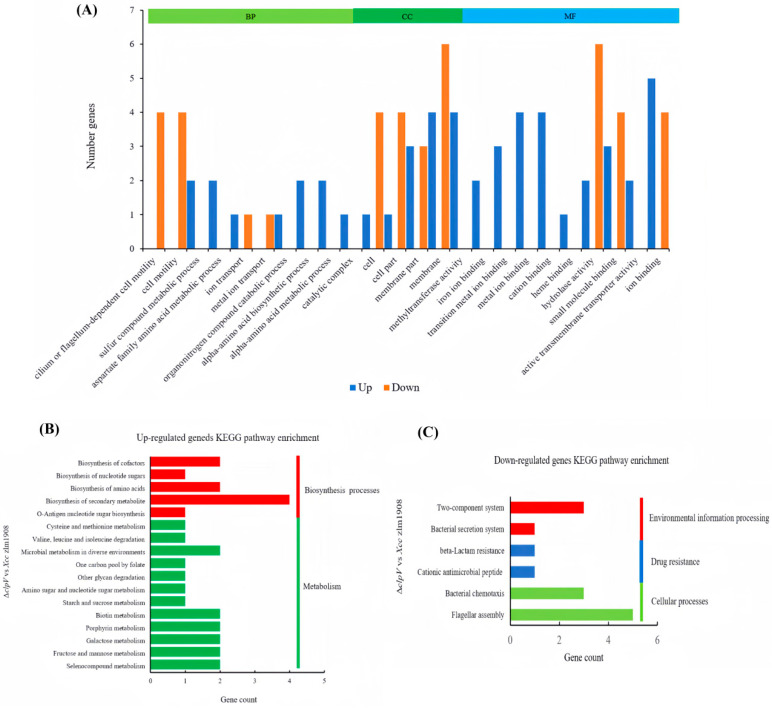
Comparison of the global transcriptomes of *Xcc* zlm1908 and Δ*clpV* by RNA-Seq. (**A**) A total of 76 genes were significantly differentially expressed in Δ*clpV* compared to *Xcc* zlm1908, with absolute fold change >1 and FDR < 0.05. GO Classification: A bar chart depicting the gene ontology (GO) classification of the 39 DEGs, categorized into three classes: “Cellular Component” (CC), “Molecular Function” (MF), and “Biological Process” (BP). (**B**) KEGG enrichment upregulated pathways. (**C**) KEGG enrichment downregulated pathways.

Gene ontology (GO) classification and enrichment analysis were performed based on clusters. We successfully annotated 39 DEGs and categorized them into these three classes: biological processes, cellular components, and molecular functions (Figure 5). Genes associated with functions such as cilium- or flagellum-dependent cell motility (GO:0001539), membrane (GO:0016020), methyltransferase activity (GO:0008168), ion binding (GO:0005506), and metal ion binding (GO:0046914) exhibited substantial changes in their expression levels. This suggests that the absence of *clpV* gene leads to alterations in motility, abnormal metal ion transport, and changes in hydrolase activity within the strain. Additionally, four DEGs related to motility, namely *flgG*, *fliM*, *flgB*, and *flgH* were identified, all of which exhibited downregulation in Δ*clpV* strain. These imply that ClpV may positively regulate the motility of *Xcc* zlm1908 by potentially influencing the transcription of motility-related genes.

Further analysis using KEGG pathway enrichment identified the most significant metabolic and transduction pathways for DEGs. The most representative pathways include flagellar assembly (KEGG-ID: xcm02040), two-component system (KEGG-ID: xcm02020), bacterial chemotaxis (KEGG-ID: xcm02030), selenocompound metabolism (KEGG-ID: xcm00450), fructose and mannose metabolism (KEGG-ID: xcm00051), and cysteine and methionine metabolism (KEGG-ID: xcm00270). These enriched KEGG pathways indicate that ClpV may regulate relevant phenotypic changes through these pathways. According to the RNA-seq results, the top 10 significantly upregulated genes were *hslV*, AMD14_RS15995, AMD14_RS09150, AMD14_RS13175, *bfr*, *metH*, *msrB*, AMD14_RS13675, AMD14_RS14885, and AMD14_RS02230. Conversely, the top 10 significantly downregulated genes were AMD14_RS24660, AMD14_RS04820, *mreD*, AMD14_RS14990, *fliN*, *flgH*, AMD14_RS13515, AMD14_RS00135, AMD14_RS12895, and *flgB*.

#### 3.3.3. The Validation of Transcriptome Data

**Figure 6:**
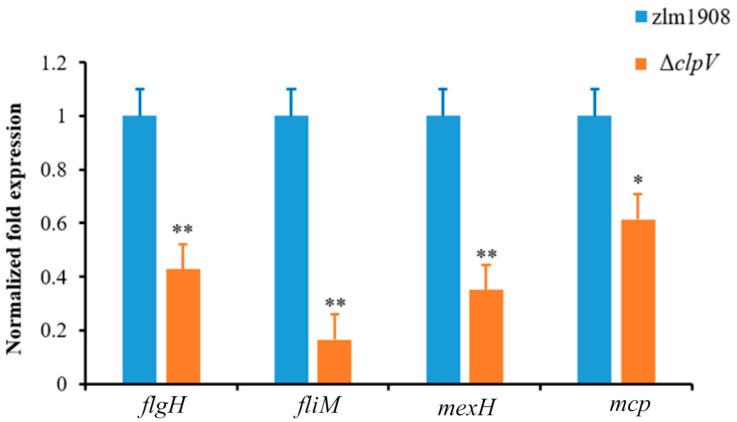
Transcription verification of RNA-seq in the *Xcc* zlm1908 and Δ*clpV* by qRT-PCR. The results are representative of three independent experiments. * *p* < 0.05; ** *p* < 0.001.

The selected genes were subjected to RT-qPCR to validate the RNA-seq data. The genes chosen for validation included *flgH*, *fliM*, *mcp*, and *mexH*. As shown in Figure 6, the results demonstrated that the expression trends of these genes were consistent with the RNA-seq data, affirming the reliability of the RNA-seq findings.

## 4. Discussion

A variety of virulence factors are present and extensively investigated in *X*. *campestris* pv. *campestris*, including commonly encoded factors such as the EPS [19], lipopolysaccharides [20], chemotaxis, flagella-driven motility, twitching motility, biofilm formation, type II secretion system (T2SS), and type III secretion system (T3SS) [21,22]. The type VI secretion system (T6SS) has been also shown to play a role in bacterial interactions, biofilm formation, interactions with eukaryotic cells, acquisition of metal ions, and responses to environmental stress in *X. campestris* pv. *campestris* [23]. Additionally, it has a significant role in the pathogenicity of *Citrobacter freundii* [24] and *X. phaseoli* pv. *manihotis* [10]. As an ATPase, the T6SS component protein ClpV (TssH) is a protein that plays a crucial role in the functioning of the T6SS, which is a complex apparatus used by various Gram-negative bacteria to transport effector proteins into target cells [25]. ClpV may utilize ATP hydrolysis to promote the unfolding of substrate proteins and serves several important roles associated with biofilm formation, chaperone activity, motility, response to stress, and aiding the secretion of effectors in *Vibrio cholerae*, *Pseudomonas aeruginosa*, and *Escherichia coli* [25,26,27]. Currently, there is limited information regarding the role of the T6SS component protein ClpV in the virulence of *Xanthomonas*.

Bacterial growth leads to increased population density, which allows bacteria to coordinate the expression of virulence factors based on their population size and enhances their ability to infect or cause disease. In our study, the results revealed no significant differences in growth between the deletion of *clpV* and wild strains during the lag phase, logarithmic phase, stationary phase, or decline phase. The deletion of *clpV* did not influence growth kinetics, indicating that the T6SS may not be essential for growth of *Xcc* zlm1908, at least in nutrient-rich environments. Biofilm formation and motility are crucial virulence factors that enable microorganisms to survive in harsh environments and infect host plants. Biofilm formation enhances bacterial resistance to both host defenses and antimicrobial agents [28]. It has been suggested that during biofilm formation, flagella serve dual functions: acting as surface adhesins and facilitating motility. In many Gram-negative bacteria, flagellar-mediated motility is essential for both initial attachment and subsequent biofilm development [29,30]. Biofilm formation assays of this study have demonstrated that the Δ*clpV* strain exhibits a reduced ability to form biofilms, aligning with previous studies on *X. phaseoli* pv. *manihotis*, *Aliivibrio avenae* subsp. *avenae*, and *Enterobacter cloacae*, where the deletion of *clpV* resulted in diminished biofilm formation [10,27,31]. Additionally, the migration patterns of *Xcc* zlm1908 and Δ*clpV* strains on swarming agar plates indicate that ClpV positively regulates the motility of *Xcc*. This finding is consistent with observations in *P. aeruginosa*, where deletion of *clpV* led to the decreased collective motility [9]. Moreover, RNA sequencing analysis revealed that the *clpV* deletion resulted in the decreased expression of four motility-related genes (*fliN*, *flgH*, *flgB*, and *fliM*) in comparison to *Xcc* zlm1908. These results underscore the critical role of ClpV in motility and flagella synthesis in *Xcc*. Moreover, the motility-related genes have a variety of further functions beyond their essential role as motility structures. They seem to contribute to biofilm formation, which seems the common mode of bacterial growth in the environment [32].

As the primary component of the biofilm matrix, EPS protect bacteria from host immune responses and antibiotic attacks, serving as a critical virulence factor for Gram-negative bacteria. By forming a protective barrier, EPS enhance bacterial survival in hostile environments, promote adhesion to surfaces, and contribute to the structural integrity of biofilms. This resilience against host defenses and antimicrobial agents underscores the importance of EPS in the pathogenicity of Gram-negative bacteria [33]. Our research shows that the Δ*clpV* produces significantly less EPS compared to *Xcc* zlm1908, suggesting that ClpV could play a vital role in the generation of extracellular polymeric substances. Some studies have indicated that when the related genes of EPS were depressed or downregulated, bacterial susceptibility to harsh environments increased [34]. There are few studies on plant-associated bacteria using the T6SS to resist abiotic stresses. The expression of T6SS-related genes has been shown to be altered under abiotic stress conditions. Although it has been speculated that this is due to the upregulation and downregulation of genes triggered by plant bacteria to resist environmental stress, the specific regulatory mechanisms remain unclear [35]. In this study, Δ*clpV* is significantly reduced in tolerance of stress (NaCl) and some antibiotics (β-lactam antibiotics and aminoglycosides), implying that ClpV could be an important factor for these processes through regulating the related proteins of EPS. Moreover, transcriptional analysis indicates that deletion of *clpV* reduces expression levels of genes associated with bacterial chemotaxis (*fliM* and *fliN*) and the efflux pump gene *mexH* limiting transport of β-lactam antibiotics to the periplasmic space, suggesting ClpV could regulate tolerance of antibiotics through multiple pathways.

In this study, the deletion of *clpV* significantly decreased the amounts of EPS, the ability of biofilm formation, adhesion on leaf surface, swarming motility, and sensitivity to stress and antibiotics in *Xcc* but did not significantly affect the pathogenicity infecting leaves of citrus hosts. This result is in agreement with findings in *Campylobacter jejuni* [7], *P. syringae* pv. *actinidiae* [36], and *Acidovorax citrulli* [37]. Some studies have shown ClpV is a crucial virulence factor, and *clpV*-deletion resulted in a significant decrease in pathogenicity in *P. plecoglossicida*, *Salmonella enterica* serovar *Typhimurium*, and *E. coli* infecting animals [38,39,40]. Those results suggest ClpV could play versatile roles in different bacterial species.

Interestingly, RNA-seq analysis revealed that the deletion of *clpV* did not affect the expression of other core genes in the T6SS. Furthermore, there was no impact on the expression of core genes in the T1SS, T2SS, and T3SS. This suggests that the ClpV in *Xcc* may primarily be involved in bacterial competition rather than in pathogenic effects on hosts. The σ54 factor serves as a multifunctional regulatory element in various vital biological processes and plays a crucial role in governing a range of phenotypic traits. It has been shown to regulate important characteristics, including motility, biofilm formation, and antibiotic resistance [41]. RNA-seq sequencing revealed a significant downregulation of the σ^54^ transcription regulator (AMD14_RS04820) in *clpV*-deletion strain. The abnormal expression of σ^54^ may lead to decreased virulence factors, such as motility, biofilm formation, EPS production, and resistance to various stresses [42]. Transcriptomic data also indicate that the absence of *clpV* impacts several metabolic processes, including phosphate transport, lactose metabolism, starch and sucrose metabolism, and selenium compound metabolism.

## 5. Conclusions

In conclusion, the results of this study suggest that ClpV negatively impacts motility, biofilm formation, extracellular polymeric substance (EPS) production, and stress resistance. The RNA-seq data indicate that ClpV is associated with bacterial chemotaxis and flagellum assembly.

## Figures and Tables

**Table 1:** Strains and plasmids used in this study.

Strains and Plasmids	Relevant Characteristic	Source or Reference
*Xcc* strains		
zlm1908	Wild type, isolated from the leaf of diseased *Citrus sinensis* ‘hongjiang’	Stored in our lab
Δ*clpV*	zlm1908, in-frame deletion of *clpV*_51-1432_	This study
C-*clpV*	Δ*clpV* with *clpV*-pBAD33CM-rp4 Recombinant vector	This study
*E. coli* strains		
DH5α	Competent cell	Takara
β2163	Competent cell	Takara
Plasmids		
pLP12	Suicide vector, Cm^+^	This study
pBAD33	Suicide vector, Cm^+^	This study
pMD18-T	Cloning vector, Amp^+^	Takara

**Table 2:** The primers used in this study.

Primer Name	Primer Sequence (5′-3′)
Primers for construction of the *clpV* deletion mutant	
*clpV*-CF/CR	TGTGGAATTGGTGCATTGGC/TGCATCGCGAGATCACTAGC
*clpV*-F/R	ACCGCATGCTGAGCCTGTTC/CAAGGGTTCATTAACTCGGG
*clpV*-F/R	ACCGCATGCTGAGCCTGTTC/CAAGGGTTCATTAACTCGGG
*clpV*-UF	GGAATCTAGACCTTGAGTCGCGAGCGACCAGTTCCGTGCG
*clpV*-UR	CATAGGTTGCCATTCTTAGTGAACAGGGAGCTGCGGGAGA
*clpV*-DF	TCTCCCGCAGCTCCCTGTTCACTAAGAATGGCAACCTATG
*clpV*-DR	ACAGCTAGCGACGATATGTCTCAAAGACCAGACCCCAGAC
*pLP*-UF/UR	GACACAGTTGTAACTGGTCCA/CAGGAACACTTAACGGCTGAC
*pLP*-UTF/UTR	CAGGAACACTTAACGGCTGAC/CAGGAATCTAGACCTTGAGTCG
Primers for qRT-PCR	
*flgH*-F/R	TGAACCTGGTCGAAAGCACC/TTCTCAAGCACGTTCAAGCC
*fliM*-F/R	TGATCTGCTTTCCCAGGACG/TCCTGGCTGGACAGATCGTA
*mcp*-F/R	AGTCGCTCATCACGTCCATC/CAGGAACACTTAACGGCTGAC
*mexH*-F/R	CAGCACCTACGTCAACGACT/AATGGTCGATGTCCTGTGGG

**Table 3:** The lesion area of the leaves at 15 days post inoculation (mm^2^).

OD_600_	Pinprick			Infiltration		
	Δ*clpV*	WT	C-*clpV*	Δ*clpV*	WT	C-*clpV*
0.1	5.24 ± 0.01	5.26 ± 0.01	5.23 ± 0.01	75.47 ± 2.15	76.19 ± 3.32	78.32 ± 3.65
0.2	5.56 ± 0.02	5.62 ± 0.03	5.57 ± 0.02	79.32 ± 6.12	83.19 ± 4.65	84.33 ± 4.76
0.5	5.44 ± 0.02	5.65 ± 0.03	5.54 ± 0.03	80.32 ± 6.43	83.16 ± 7.21	82.76 ± 4.68
0.7	5.97 ± 0.03	5.92 ± 0.04	5.88 ± 0.02	83.43 ± 7.53	82.65 ± 8.32	89.54 ± 8.32
0.9	6.09 ± 0.04	6.24 ± 0.04	6.13 ± 0.05	85.43 ± 9.02	82.14 ± 10.32	86.45 ± 9.46

## Data Availability

The raw data supporting the conclusions of this article will be made available by the authors on request.

## Supplementary Materials

The following supporting information can be downloaded at: https://www.mdpi.com/article/10.3390/microorganisms12122536/s1, Table S1: RNA-sequencing analysis of differentially expressed genes in Δ*clpV* vs *Xcc* zlm1908.
