# Supplementary material for: The Role of ClpV in the Physiology and Pathogenicity of Xanthomonas citri subsp. citri Strain zlm1908
Source: Microorganisms. 2024 Dec 9;12(12):2536. doi: 10.3390/microorganisms12122536 (PMC11677086; doi:10.3390/microorganisms12122536)
Supplement: Supplementary file 1 [file microorganisms-12-02536-s001.zip › microorganisms-3304200-supplementary.pdf]

Table S1 RNA-sequencing analysis of differentially expressed genes in Δ*clpV* vs *Xcc* zlm1908

gene_id	gene_name	gene_description	log_2FoldChange	*padj*
Up-regulated genes				
AMD14_RS03780	hslV	ATP-dependent protease subunit HslV	1.073816003	5.67E-23
AMD14_RS15995	AMD14_RS15995	TonB-dependent receptor	1.136668266	5.84E-23
AMD14_RS09150	AMD14_RS09150	LLM class flavin-dependent oxidoreductase	1.515839163	1.09E-21
AMD14_RS13175	AMD14_RS13175	fructose-specific PTS transporter subunit EIIC	1.611231424	2.09E-20
AMD14_RS03035	bfr	bacterioferritin	1.089164879	9.59E-19
AMD14_RS08430	metH	methionine synthase	1.106421177	3.18E-18
AMD14_RS19080	msrB	MsrB	1.006726069	6.87E-18
AMD14_RS13675	AMD14_RS13675	glycoside hydrolase family 97 protein	1.012802098	3.25E-14
AMD14_RS14885	AMD14_RS14885	efflux RND transporter permease subunit	1.213114845	3.62E-14
AMD14_RS02230	AMD14_RS02230	methionine synthase	1.220790562	6.94E-12
AMD14_RS07120	AMD14_RS07120	RNA-binding S4 domain-containing protein	1.011293928	4.70E-09
AMD14_RS19085	AMD14_RS19085	MliC family protein	1.036488088	8.34E-09
AMD14_RS13170	pfkB	1-phosphofructokinase	1.18238541	1.02E-07
AMD14_RS15910	AMD14_RS15910	alpha/beta hydrolase	1.740035583	4.19E-07
AMD14_RS21310	AMD14_RS21310	GlsB/YeaQ/YmgE family stress response membrane protein	1.040502169	2.96E-06
AMD14_RS16000	AMD14_RS16000	alpha-L-fucosidas	1.020915139	4.56E-06
AMD14_RS14590	AMD14_RS14590	Domain of unknown function	1.057518888	1.02E-05
AMD14_RS08445	AMD14_RS08445	acyl-CoA dehydrogenase family protein	1.146631826	1.17E-05
AMD14_RS02120	AMD14_RS02120	hypothetical protein	1.189651793	6.73E-05
AMD14_RS02235	kdgT	2-keto-3-deoxygluconate transporter(kdgT)	1.560114858	7.66E-05
AMD14_RS06515	AMD14_RS06515	alpha-ketoglutarate-dependent dioxygenase AlkB	1.112412428	0.000869
AMD14_RS16475	AMD14_RS16475	cytochrome P45	1.844750156	0.00305
AMD14_RS12035	AMD14_RS12035	NAD(P)-dependent oxidoreductase	2.165620365	0.005813

AMD14_RS09110	AMD14_RS09110	Class I SAM-dependent methyltransferase	4.470081042	0.005872
AMD14_RS23495	AMD14_RS23495	hypothetical protein	1.151434197	0.022601
AMD14_RS25415	AMD14_RS25415	tetratricopeptide repeat protein	1.674212346	0.027434
AMD14_RS09705	AMD14_RS09705	tryptophan-rich sensory protein	1.076751021	0.029012
AMD14_RS21165	tssF	tssF	1.944273393	0.029366
AMD14_RS16780	AMD14_RS16780	DNA-binding protein	1.08946672	0.035902
AMD14_RS23370	AMD14_RS23370	SAM-dependent methyltransferas	2.845769395	0.041887
AMD14_RS24985	AMD14_RS24985	hypothetical protein	1.217034253	0.042642
AMD14_RS12800	AMD14_RS12800	Tn3 family transposase	1.84611689	0.043901
AMD14_RS19305	galE	UDP-glucose 4-epimerase GalE	1.024664467	0.04605
AMD14_RS19490	AMD14_RS19490	hypothetical protein	3.691357637	0.049122
Down-regulated genes				
AMD14_RS17475	fhuE	ferric-coprogen receptor FhuE	-3.399394	1.14E-63
AMD14_RS24605	AMD14_RS24605	hypothetical protein	-1.313208	5.67E-23
AMD14_RS01840	AMD14_RS01840	S9 family peptidases	-1.130003	5.84E-23
AMD14_RS21280	AMD14_RS21280	Bacterial protein of unknown function	-1.233489	8.42E-22
AMD14_RS14815	AMD14_RS14815	TonB-depende receptor	-1.188261	1.09E-21
AMD14_RS04625	AMD14_RS04625	prolyl oligopeptidase family serine peptidase	-1.016189	2.96E-21
AMD14_RS00610	AMD14_RS00610	hypothetical protein	-1.04451	4.04E-21
AMD14_RS01890	AMD14_RS01890	DUF4198 domain-containing protein	-1.562785	2.09E-20
AMD14_RS09185	AMD14_RS09185	S10 family peptidase	-1.25644	9.59E-19
AMD14_RS10945	AMD14_RS10945	efflux RND transporter permease subunit	-1.190374	3.18E-18
AMD14_RS09470	AMD14_RS09470	TonB-dependent receptor	-1.231039	6.87E-18
AMD14_RS07585	AMD14_RS07585	ABC transporter ATP-binding protein	-1.115899	1.97E-16
AMD14_RS10940	AMD14_RS10940	MexH family multidrug efflux RND transporter periplasmic adaptor	-1.300861	4.34E-16
AMD14_RS15340	AMD14_RS15340	TonB-dependent siderophore receptor	-1.658613	2.91E-15
AMD14_RS07820	AMD14_RS07820	TonB-dependent siderophore receptor	-1.659324	3.78E-15
AMD14_RS09885	AMD14_RS09885	ferrous iron transporter	-1.030893	6.02E-15

AMD14_RS21155	tssH	tssH	-6.319605	3.25E-14
AMD14_RS17085	AMD14_RS17085	CPBP family intramembrane metalloprotease	-1.359219	3.62E-14
AMD14_RS16845	AMD14_RS16845	Pilin (bacterial filament)	-1.722352	5.42E-14
AMD14_RS01385	AMD14_RS01385	TonB-dependent siderophore receptor	-1.511729	6.94E-12
AMD14_RS19420	AMD14_RS19420	hypothetical protein	-1.027142	1.04E-11
AMD14_RS04705	AMD14_RS04705	hemin uptake protein HemP	-1.904854	3.11E-11
AMD14_RS01885	AMD14_RS01885	Protein of unknown function	-1.674897	1.78E-10
AMD14_RS18920	AMD14_RS18920	Protein of unknown function	-1.017748	2.65E-10
AMD14_RS18785	AMD14_RS18785	hypothetical protein	-1.005131	6.04E-10
AMD14_RS13990	AMD14_RS13990	PilW family protein	-1.041419	4.70E-09
AMD14_RS16840	AMD14_RS16840	Pilin (bacterial filament)	-1.159885	8.35E-09
AMD14_RS10520	flgG	flagellar basal-body rod protein FlgG	-1.299444	4.00E-08
AMD14_RS01880	AMD14_RS01880	PepSY-associated TM helix domain-containing protein	-1.105184	1.02E-07
AMD14_RS25200	AMD14_RS25200	AAA family ATPase	-1.078102	4.19E-07
AMD14_RS10355	fliM	flagellar motor switch protein FliM	-1.259302	1.44E-06
AMD14_RS00280	AMD14_RS00280	hypothetical protein	-2.048312	2.96E-06
AMD14_RS10545	flgB	flagellar basal body rod protein FlgB	-3.404886	3.27E-06
AMD14_RS12895	AMD14_RS12895	methyl-accepting chemotaxis protein	-1.120995	4.56E-06
AMD14_RS00135	AMD14_RS00135	Tn3-like element ISXc4 family transposase	-2.534272	4.80E-06
AMD14_RS13515	AMD14_RS13515	hypothetical protein	-1.153045	1.02E-05
AMD14_RS10515	flgH	flagellar basal body L-ring protein FlgH	-1.252387	1.13E-05
AMD14_RS10350	fliN	flagellar motor switch protein FliN	-1.660455	1.17E-05
AMD14_RS14990	AMD14_RS14990	STAS domain-containing protein	-3.811261	6.39E-05
AMD14_RS03890	mreD	rod shape-determining protein MreD	-1.062411	6.73E-05
AMD14_RS04820	AMD14_RS04820	sigma-54 dependent transcriptional regulator	-1.397738	7.66E-05
AMD14_RS24660	AMD14_RS24660	hypothetical protein	-1.285914	0.000101
